# MDA-7/IL-24 Induces Bcl-2 Denitrosylation and Ubiquitin-Degradation Involved in Cancer Cell Apoptosis

**DOI:** 10.1371/journal.pone.0037200

**Published:** 2012-05-21

**Authors:** Hui Tian, Jing Wang, BaoFu Zhang, JieHui Di, FeiFei Chen, HuiZhong Li, LianTao Li, DongSheng Pei, JunNian Zheng

**Affiliations:** Laboratory of Biological Cancer Therapy, Xuzhou Medical College, Xuzhou, People's Republic of China; Massachusetts Eye & Ear Infirmary-Harvard Medical School, United States of America

## Abstract

MDA-7/IL-24 was involved in the specific cancer apoptosis through suppression of Bcl-2 expression, which is a key apoptosis regulatory protein of the mitochondrial death pathway. However, the underlying mechanisms of this regulation are unclear. We report here that tumor-selective replicating adenovirus ZD55-IL-24 leads to Bcl-2 S-denitrosylation and concomitant ubiquitination, which take part in the 26S proteasome degradation. IL-24-siRNA completely blocks Bcl-2 ubiquitination via reversion of Bcl-2 S-denitrosylation and protects it from proteasomal degradation which confirmed the significant role of MDA-7/IL-24 in regulating posttranslational modification of Bcl-2 in cancer cells. Nitric oxide (NO) is a key regulator of protein S-nitrosylation and denitrosylation. The NO donor, sodium nitroprusside (SNP), down-regulates Bcl-2 S-denitrosylation, attenuates Bcl-2 ubiquitination and subsequently counteracts MDA-7/IL-24 induced cancer cell apoptosis, whereas NO inhibitor 2-(4-carboxyphenyl)-4,4,5,5-tetramethylimidazoline-1-oxy-3-oxide (PTIO) shows the opposite effect. At the same time, these NO modulators fail to affect Bcl-2 phosphorylation, suggesting that NO regulates Bcl-2 stability in a phosphorylation-independent manner. In addition, Bcl-2 S-nitrosylation reduction induced by ZD55-IL-24 was attributed to both iNOS decrease and TrxR1 increase. iNOS-siRNA facilitates Bcl-2 S-denitrosylation and ubiquitin-degradation, whereas the TrxR1 inhibitor auranofin prevents Bcl-2 from denitrosylation and ubiquitination, thus restrains the caspase signal pathway activation and subsequent cancer cell apoptosis. Taken together, our studies reveal that MDA-7/IL-24 induces Bcl-2 S-denitrosylation via regulation of iNOS and TrxR1. Moreover, denitrosylation of Bcl-2 results in its ubiquitination and subsequent caspase protease family activation, as a consequence, apoptosis susceptibility. These findings provide a novel insight into MDA-7/IL-24 induced growth inhibition and carcinoma apoptosis.

## Introduction

Interleukin 24(IL-24), also called melanoma differentiation associated gene-7(MDA-7), is a unique member of the IL-10 gene family, that displays a selective induction of cancer specific apoptosis without deleterious effects on the normal cells [1]–[3]. MDA-7/IL-24 induces growth suppression and apoptosis in a broad spectrum of human cancer cells, including melanoma, malignant glioma, and carcinomas of the breast [4]–[8]. The involvement of MDA-7/IL-24-induced apoptosis in tumor tissues was associated with endoplasmic reticulum (ER) stress and mitochondrial dysfunction and reactive oxygen species (ROS) production [7], [9], [10]. Moreover, MDA-7/IL-24 induced potent “bystander antitumor” activity, an ability to block tumor angiogenesis, synergy with radiation, chemotherapy, monoclonal antibody therapies and immune modulatory activity [11], [12], which make it a ideal tool for cancer gene therapy.

Although the pathways by which MDA-7/IL-24 enhances apoptosis in tumor cells are not fully elucidated, evidence from several studies suggests that MDA-7/IL-24 mediates many proteins important for the onset of growth inhibition and involvement of the mitochondrial apoptotic cell death pathway [7]. B-cell lymphoma gene 2(Bcl-2), one of the anti-apoptotic Bcl-2-family members, is localized in the outer mitochondrial membrane. Some antiapoptotic mechanisms of Bcl-2 include regulation of calcium homeostasis and neutralization of proapoptotic protein Bax by forming heterodimers. In addition, Bcl-2 promoted the blockade of cytochrome c release and the association with mitochondrial apoptosis factor Apaf1, finally prevented the activation of caspase protease family and preserved mitochondrial integrity [13], [14]. MDA-7/IL-24 repressed Bcl-2 protein expression, which thus increased the ratio of specific pro- and anti-apoptotic proteins tilting the balance from survival to death in carcinoma cells. In contrast, overexpression of Bcl-2 protected prostate cancer cells from MDA-7/IL-24-mediated apoptosis, suggesting Bcl-2 plays an important role in cancer cell apoptosis in response to MDA-7/IL-24 [8]. However, the exact mechanism by which MDA-7/IL-24 regulated Bcl-2 to facilitate the mitochondrial dysfunction has not been identified. In the present study, we used tumor-selective replicating adenovirus expressing IL-24 (ZD55-IL-24) which deleted the essential viral E1B 55 kDa gene and exerted a strong cytopathic effect and significant apoptosis in tumor cells without normal cells [15] to further explore the mechanism of MDA-7/IL-24 inducing Bcl-2 down-regulation and subsequent carcinoma cell apoptosis.

**Figure 1:**
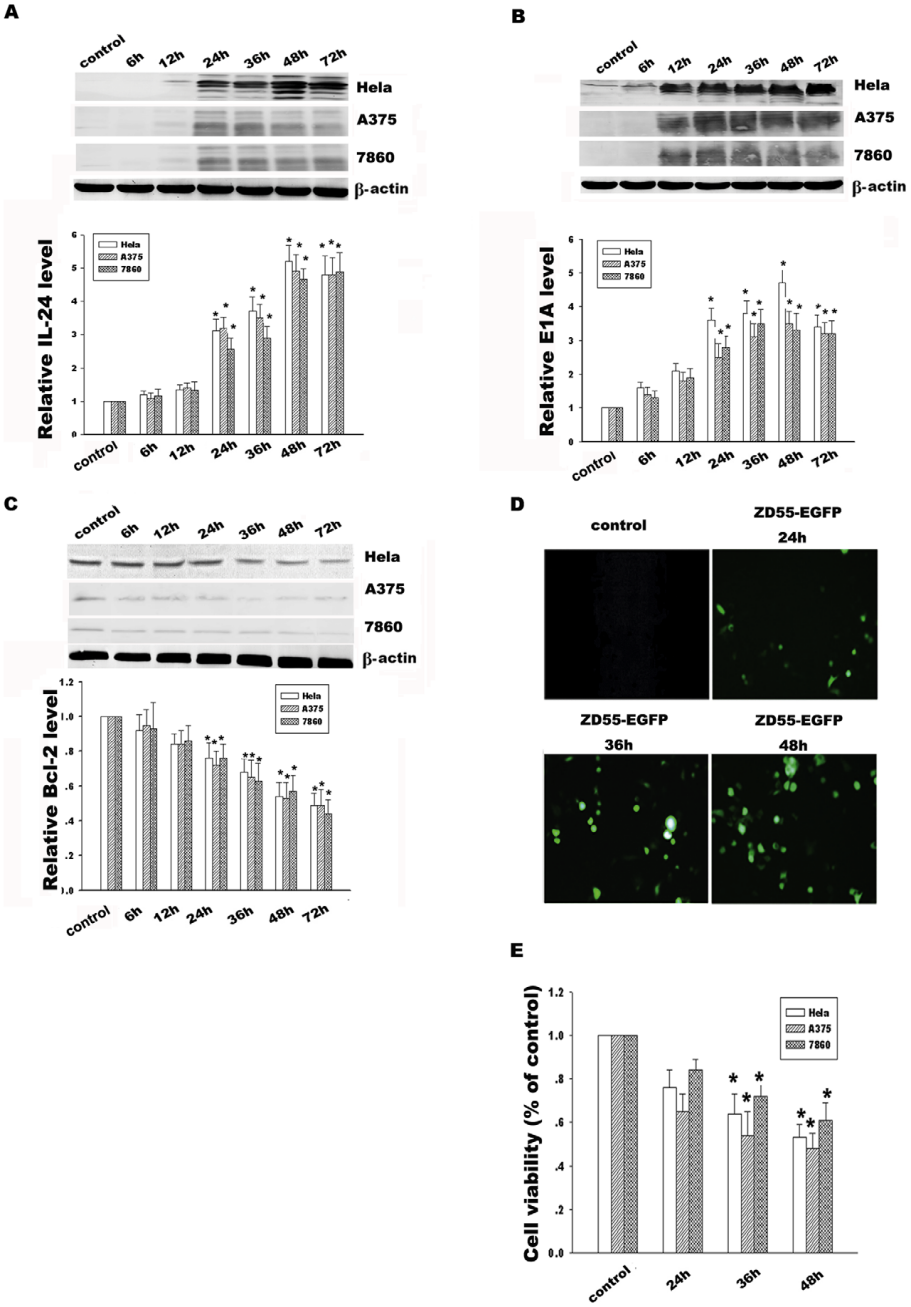
ZD55-IL-24 mediated IL-24, E1A and Bcl-2 expression in Hela, A375 and 7860 cells. (**A**) Time course analysis of IL-24 protein expression in Hela, A375 and 7860 cells treated with ZD55-IL-24 (20 MOI). (**B**) Time course analysis of E1A protein expression in Hela, A375 and 7860 cells treated with ZD55-IL-24(20 MOI). (**C**) Time course analysis of Bcl-2 protein expression in Hela, A375 and 7860 cells treated with ZD55-IL-24(20 MOI). β-actin was used as a loading control. (**D**) ZD55-EGFP (5 MOI) carrying report gene EGFP was used to detect infection efficiency of the replicative adenovirus at different time points (Magnification×200). (**E**) Hela, A375 and 7860 cells viability treated with ZD55-IL-24(20 MOI) at the different time points were determined by MTT assay. Data are means±standard deviation (S.D.) from three independent experiments (n = 3); *p<0.05 versus control group.

**Figure 2:**
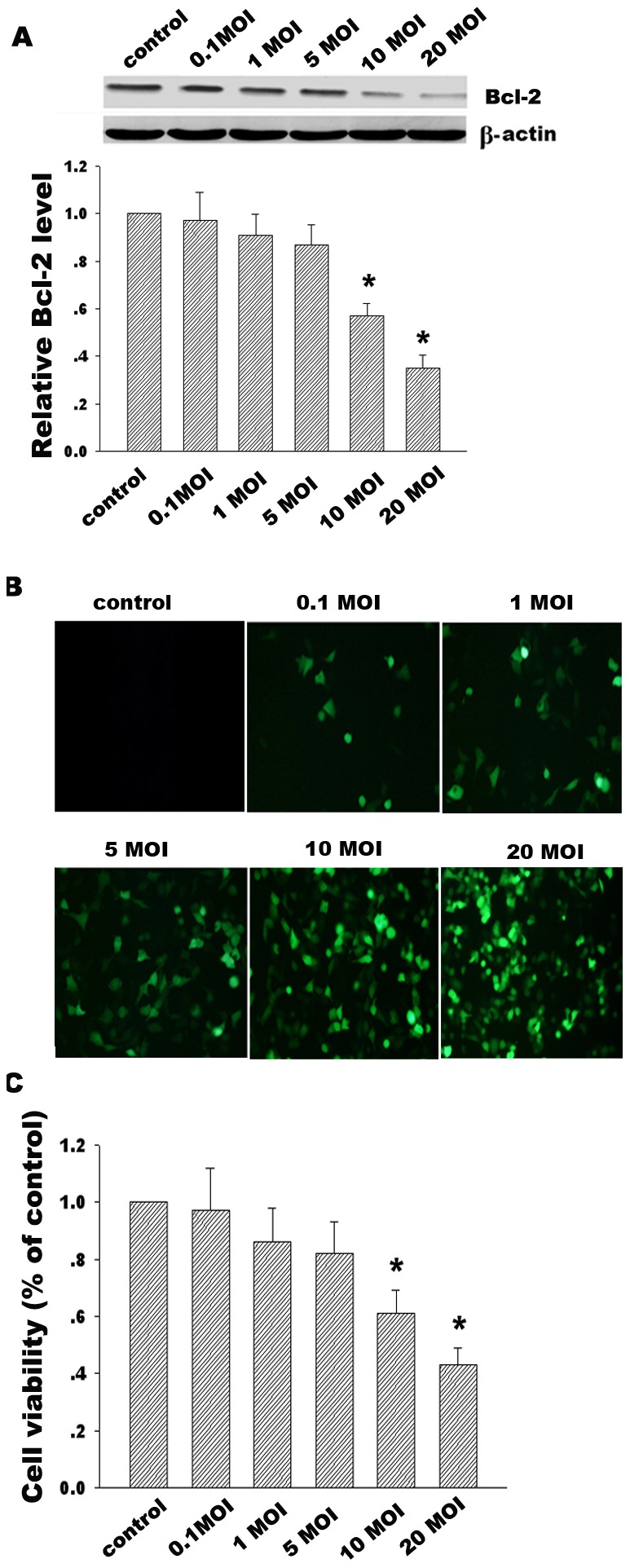
Effect of different titers of ZD55-IL24 on Bcl-2 expression and Hela cells viability. (**A**) Effects of the different titers of ZD55-IL-24 (0.1 MOI, 1 MOI, 5 MOI, 10 MOI, 20 MOI) on Bcl-2 expression induced by Western blotting 48 h after infection of ZD22-IL24. β-actin was used as a loading control. (**B**) ZD55-EGFP at the different titers was used to detect infection efficiency of replicative adenovirus (Magnification×200). (**C**) Hela cell viability treated with the different titers of ZD55-IL-24 was determined by MTT assay. Data are means±standard deviation (S.D.) from three independent experiments (n = 3); *p<0.05 versus control group.

**Figure 3:**
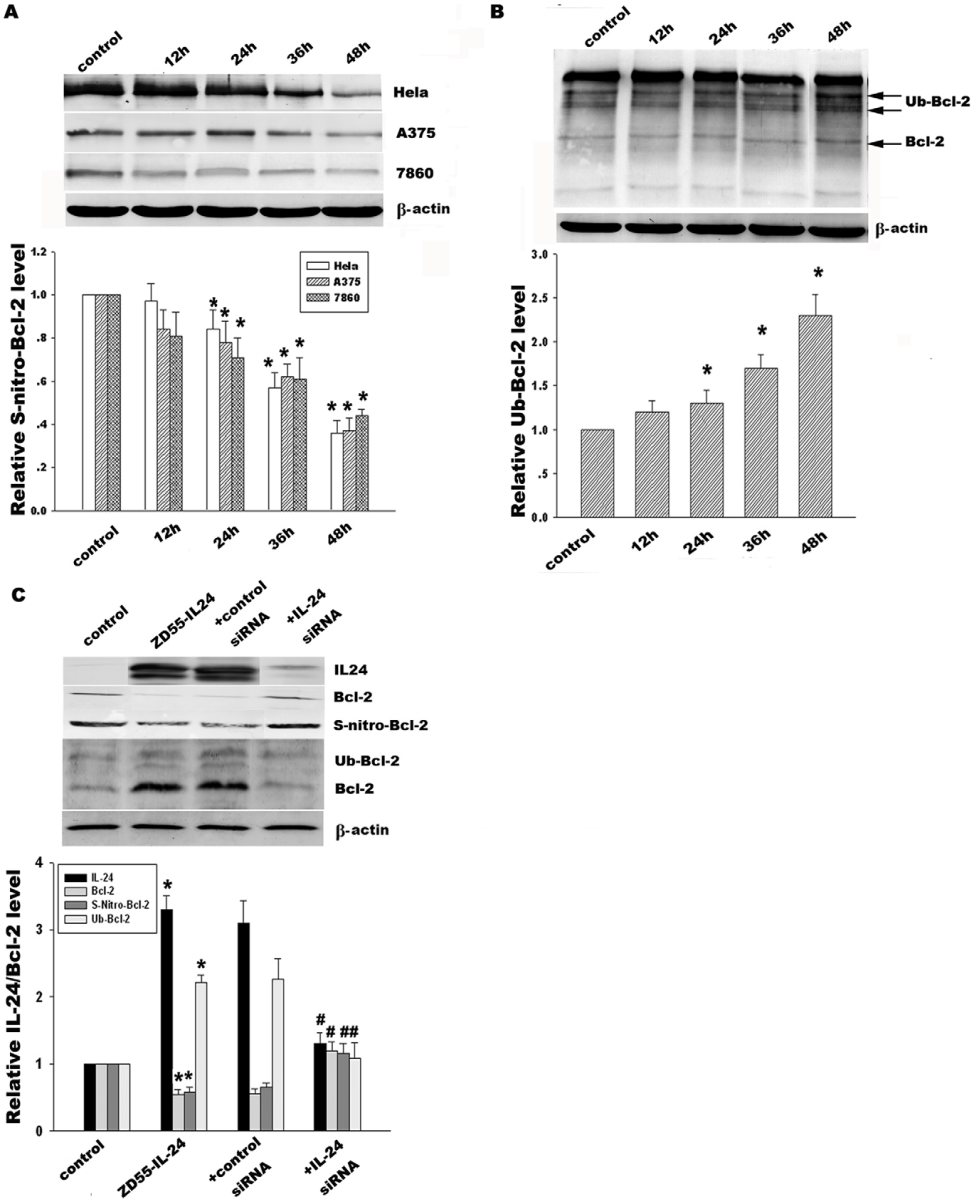
Effect of ZD55-IL-24 on Bcl-2 S-nitrosylation and ubiquitination. (**A**) Hela, A375 and 7860 cells in response to ZD55-IL-24 (20 MOI) were prepared for immunoprecipitation using anti-Bcl-2 antibody. The resulting immune complexes were analyzed for anti-S-nitrosocysteine by Western blotting and stood for Bcl-2 S-nitrosylation level at the different time points 12 h, 24 h, 36 h and 48 h, respectively. (**B**) Time course of Bcl-2 ubiquitination in Hela cells was detected by immunoprecipitation using anti-Bcl-2 antibody and then followed by immunoblotting with anti-ubiquitin antibody. (**C**) Effect of IL-24-siRNA (100 nM) upon IL-24, Bcl-2 expression, Bcl-2 S-nitrosylation and ubiquitination in Hela cells was detected by Western blotting and co-immunoprecipitation. β-actin was used as a loading control. The corresponding bands were scanned and the optical density (O.D.) was determined as the fold change versus control group. Data are means±standard deviation (S.D.) from three independent experiments (n = 3); *p<0.05 versus control group; ^#^ *p*<0.05 versus scrambled siRNA group.

**Figure 4:**
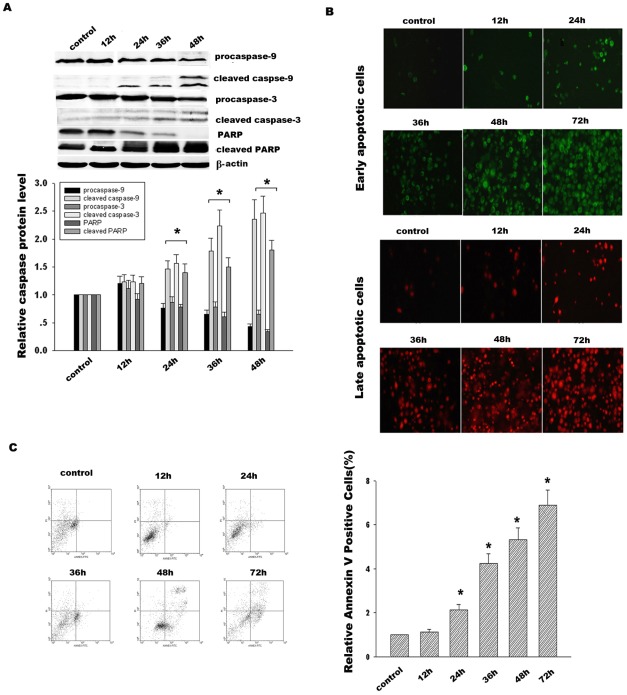
Effect of ZD55-IL-24 on activation of caspase signal pathway and cancer cell apoptosis. (**A**) Time course analysis of caspase-9, caspase-3 and PARP in Hela cells treated with ZD55-IL-24 (20 MOI). The corresponding bands were scanned and the optical density (O.D.) was determined as the fold change versus control group. (**B**) Early apoptosis and late apoptosis in Hela cells were detected by staining cells with Annexin V-FITC (green color) and Propidium Iodide (red color) at the different time points. (**C**) Hela cells treated with ZD55-IL-24 for the different time were stained with Annexin V-FITC/Propidium Iodide (PI) and immediately analyzed by flow cytometry. Data are presented as the percentage of Annexin V positive cells from three independent experiments. *p<0.05 versus control group.

**Figure 5:**
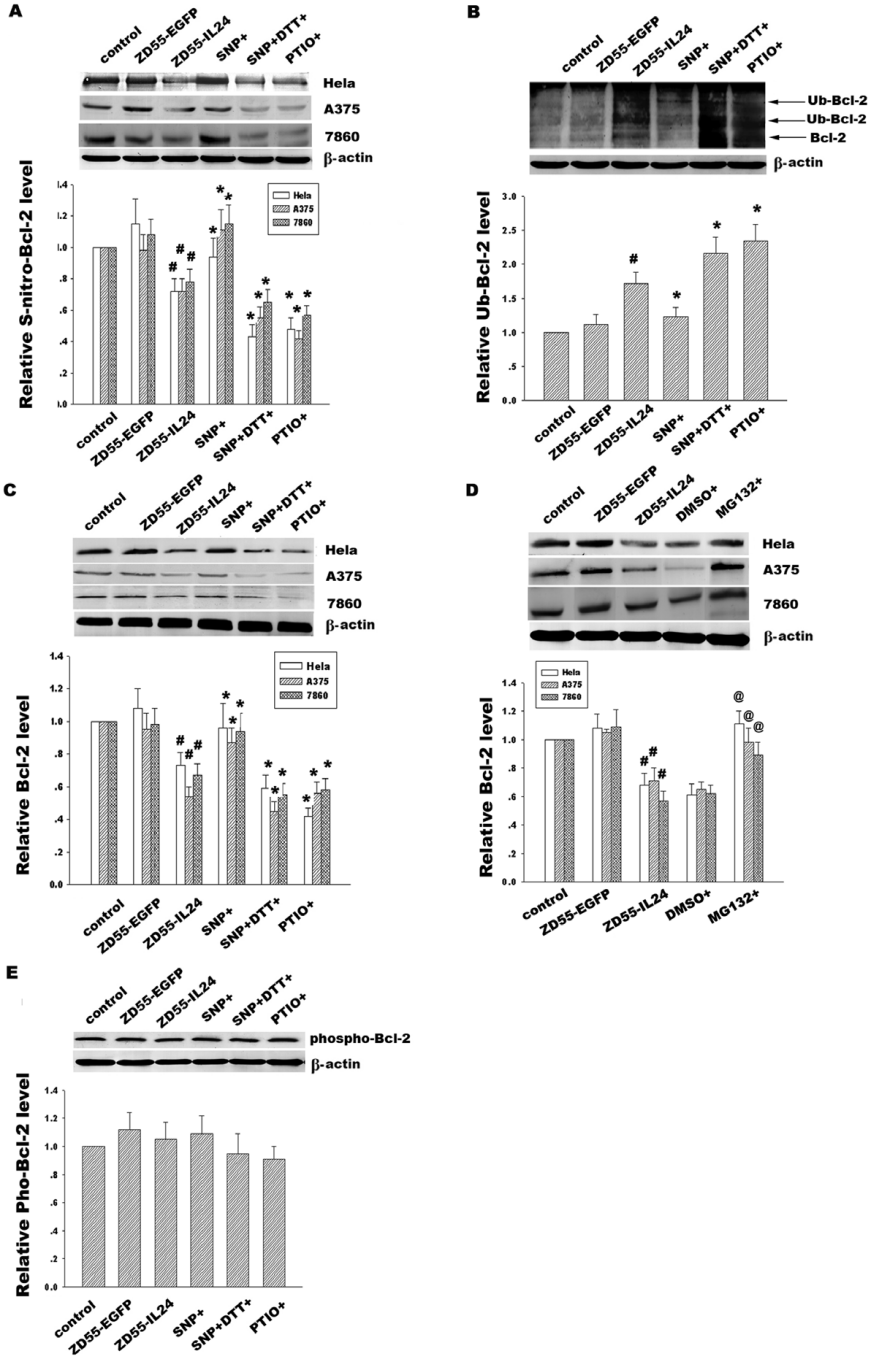
Effect of NO modulators on Bcl-2 S-nitrosylation, ubiquitination and protein expression. (**A**) Hela, A375 and 7860 cells were pretreated with ZD55-IL-24 (20 MOI) for 24 h and then treated with NO donor SNP(2 mM), NO inhibitor PTIO(300 µM) and SNP co-administration of reducing agent DTT(10 mM) for 6 h respectively. Bcl-2 S-nitrosylation was detected by immunoprecipitation using anti-Bcl-2 antibody and then followed by immunoblotting with anti-S-nitrosocysteine antibody. (**B**) Effect of NO modulators on Bcl-2 ubiquitination in Hela cells were detected by immunoprecipitation using anti-Bcl-2 antibody and then followed by immunoblotting with anti-ubiquitin antibody. (**C**) Effect of NO modulators on Bcl-2 expression in Hela, A375 and 7860 cells was detected by western blotting using anti-Bcl-2 antibody. (**D**) Hela, A375 and 7860 cells were pretreated with ZD55-IL24 for 24 h and then treated with proteasomal inhibitor MG132 (10 µM) for 6 h. Bcl-2 expression was analyzed by Western blotting using anti-Bcl-2 antibody. (**E**) Bcl-2 phosphorylation in Hela cells was detected by Western blotting with anti-p-Bcl-2(Ser87) antibody. β-actin was used as a loading control. The corresponding bands were scanned and the intensities were determined by optical density (O.D) measurements. Data are means±standard deviation (S.D.) from three independent experiments (n = 3). ^#^ *p*<0.05 versus ZD55-EGFP; *p<0.05 versus ZD55-IL-24 group; ^@^ *p*<0.05 versus DMSO group.

**Figure 6:**
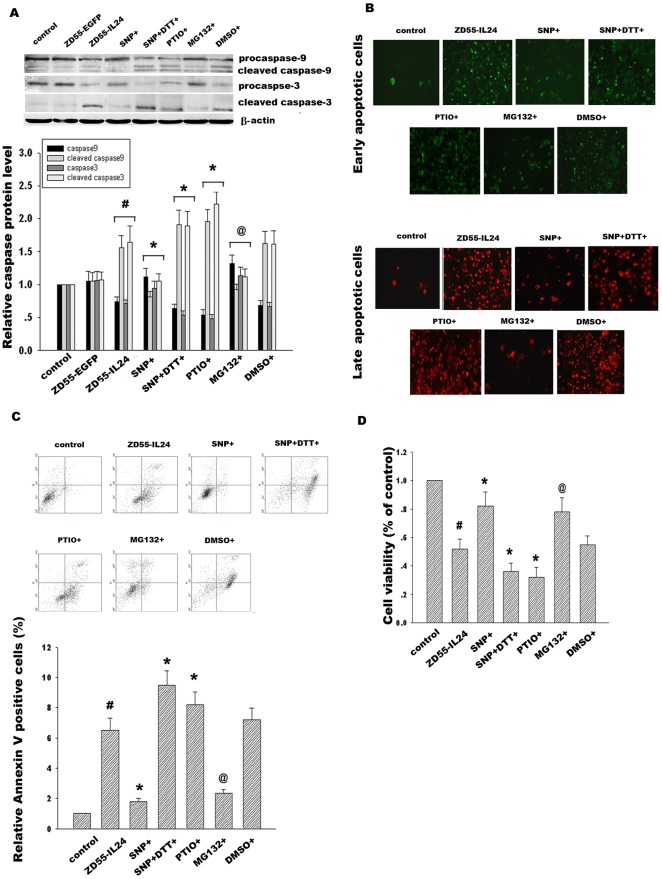
Effect of NO modulators on caspase signal pathway and Hela cell viability. (A) Hela cells were pretreated with ZD55-IL24 (20 MOI) for 24 h and then added with NO donor SNP(2 mM), NO inhibitor PTIO(300 µM), SNP co-administration of reducing agent DTT(10 mM) and proteasomal inhibitor MG132(10 µM) for 6 h respectively. Procaspase-9, cleaved caspase-9, procaspase-3, cleaved caspase-3 were detected by Western blotting. (B) Effect of NO modulators SNP, PTIO, SNP+DTT and proteasome inhibitor MG132 on early apoptosis and late apoptosis in Hela cells treated with ZD55-IL-24 were detected by staining with Annexin V-FITC (green color) and Propidium Iodide (red color) respectively. (C) Hela cells were stained with Annexin V-FITC and Propidium Iodide and immediately analyzed by flow cytometry. (D) Effects of NO modulators and MG132 on Hela cell viability were determined by MTT assay. The corresponding bands were scanned and the optical density (O.D.) was determined as the fold change versus control group. Data are means±standard deviation (S.D.) from three independent experiments (n = 3). ^#^ *p*<0.05 versus ZD55-EGFP group; *p<0.05 versus ZD55-IL-24 group; ^@^ *p*<0.05 versus DMSO group.

**Figure 7:**
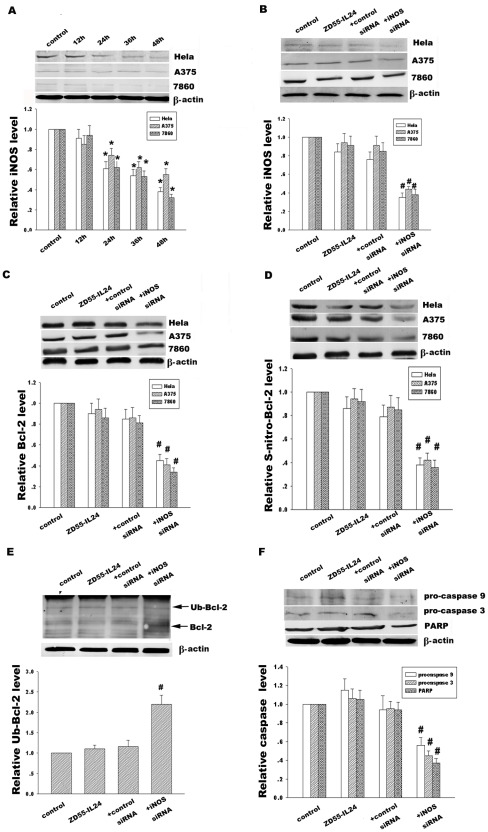
Effect of iNOS on Bcl-2 S-nitrosylaiton, ubiquitination and protein expression and caspase signal pathway. (A) Time course analysis of iNOS expression in Hela, A375 and 7860 cells treated with ZD55-IL-24 (20 MOI). (B) Hela, A375 and 7860 cells were transfected with iNOS-siRNA (100 nM) for 12 h, then treated with ZD55-IL-24 for 12 h. Effect of iNOS-siRNA on iNOS expression was analyzed by Western blotting. (C) Effect of iNOS-siRNA on Bcl-2 protein expression was analyzed by Western blotting. (D) Effect of iNOS-siRNA on Bcl-2 S-nitrosylation were detected by immunoprecipitation using anti-Bcl-2 antibody and then followed by immunoblotted with anti-S-nitrosocysteine antibody. (E) Effect of iNOS-siRNA on ubiquitin-Bcl-2 was detected by immunoprecipitation using anti-Bcl-2 antibody and then followed by immunoblotting with anti-ubiquitin antibody. (F) Effect of iNOS-siRNA on caspase-9, caspase-3 and PARP in Hela cells was detected by Western blotting. The corresponding bands were scanned and the optical density (O.D.) was determined as the fold change versus control group. Data are means±standard deviation (S.D.) from three independent experiments (n = 3). *p<0.05 versus control group; ^#^ *p*<0.05 versus scrambled siRNA group.

**Figure 8:**
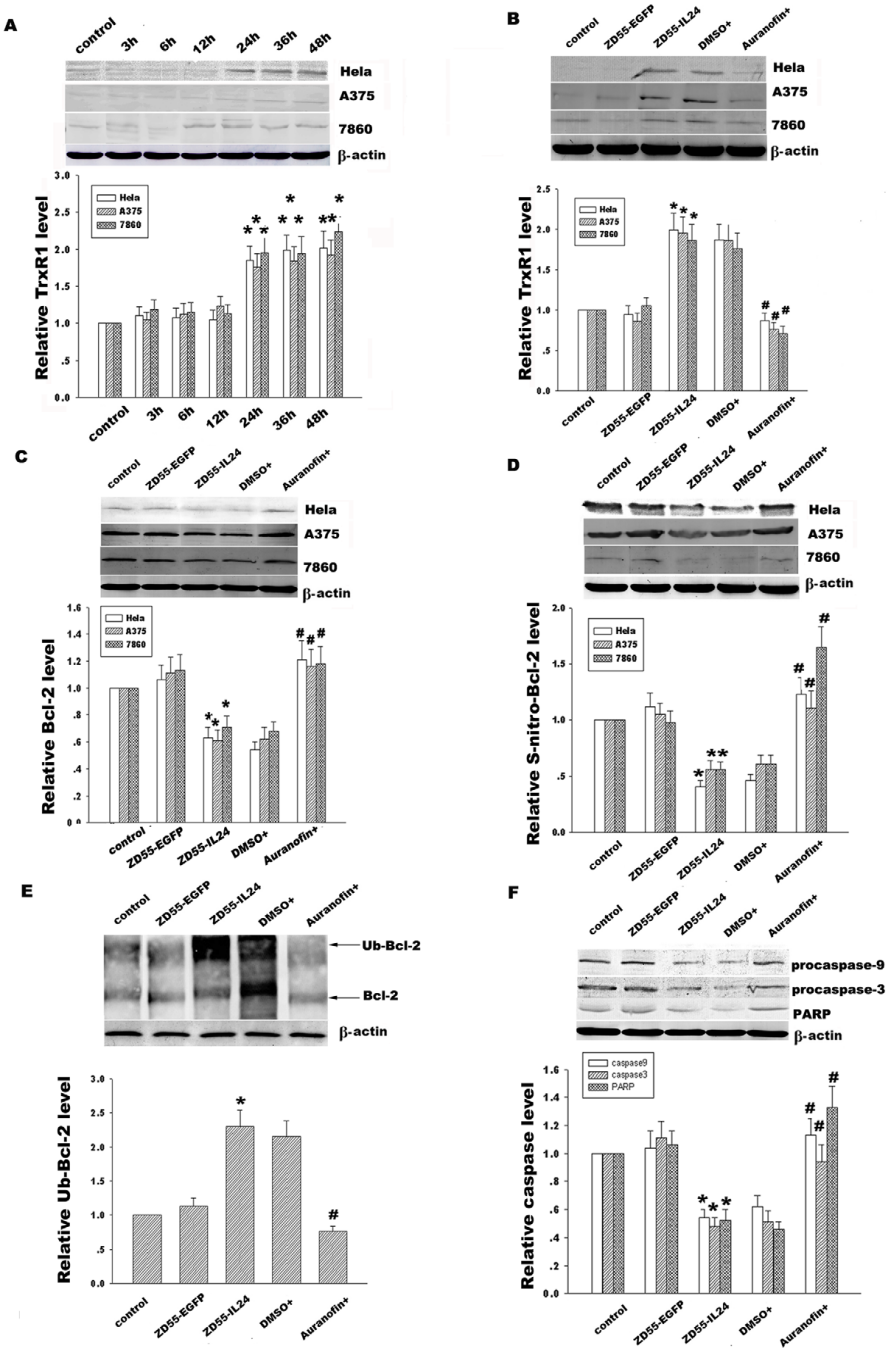
Effect of TrxR1 on Bcl-2 S-nitrosylaiton, ubiquitination and protein expression and caspase signal pathway. (**A**) Time course analysis of TrxR1 expression in Hela, A375 and 7860 cells treated with ZD55-IL-24 (20 MOI). (**B**) Hela, A375 and 7860 cells were treated with ZD55-IL-24 for 24 h and then added with TrxR1 inhibitor auranofin (5 µM) for 6 h. Effect of TrxR1 inhibitor auranofin on TrxR1 protein expression was detected by Western blotting. (C) Effect of TrxR1 inhibitor auranofin on Bcl-2 protein expression in Hela, A375 and 7860 cells was detected by the western blot assay. (D) Effect of TrxR1 inhibitor auranofin on Bcl-2 S-nitrosylation was detected by immunoprecipitation using anti-Bcl-2 antibody and then followed by immunoblotting with anti-S-nitrosocysteine antibody. (E) Effect of TrxR1 inhibitor auranofin on Bcl-2 ubiquitination in Hela cells was detected by immunoprecipitation using anti-Bcl-2 antibody and then followed by immunoblotting with anti-ubiquitin antibody. (F) Effect of TrxR1 inhibitor auranofin on caspase-9, 3 and PARP was detected by Western blotting. β-actin was used as a loading control. The corresponding bands were scanned and the optical density (O.D.) was determined as the fold change versus control group. Data are means±standard deviation (S.D.) from three independent experiments (n = 3). *p<0.05 versus ZD55-EGFP; ^#^ *p*<0.05 versus DMSO group.

**Figure 9:**
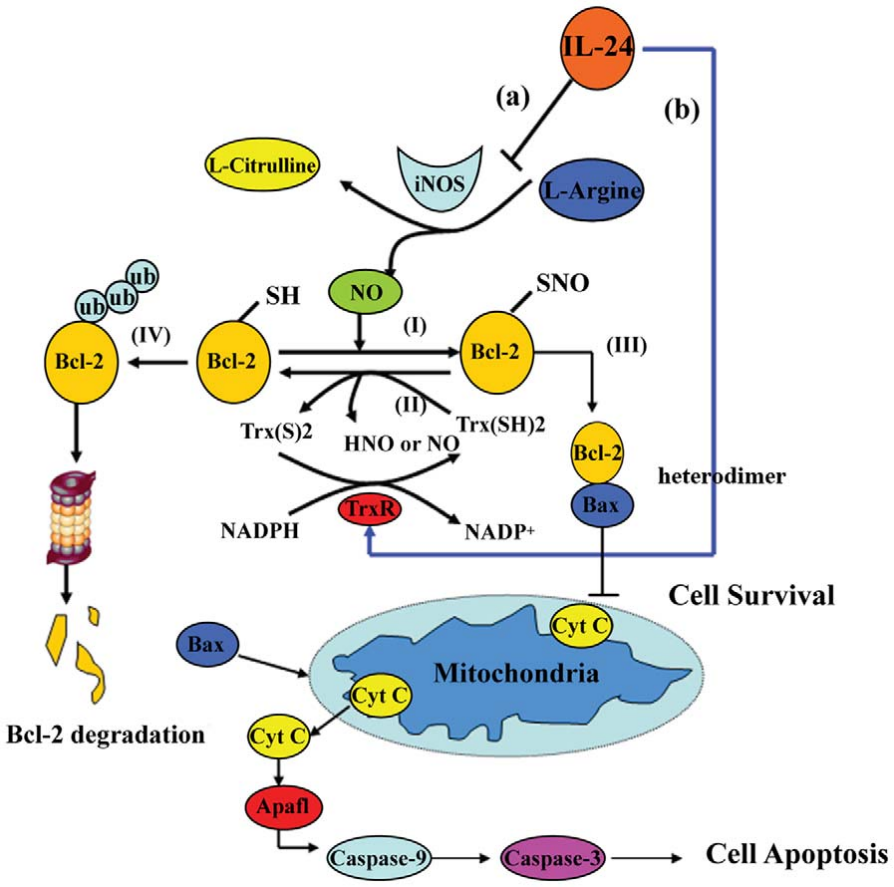
Schematic model of IL-24 induced cancer cell apoptosis. (**I**) IL-24 inhibits iNOS expression leading to reduction of NO turnover and attenuation of Bcl-2 S-nitrosylation. (**II**) Trx denitrosylates S-nitrosylated Bcl-2 through its dithiol moiety, thereby forming a reduced Bcl-2 and oxidized Trx; oxidized Trx is reduced (and therefore reactivated) by the seleno-flavoprotein Trx reductase(TrxR) and NADPH, suggesting that TrxR through reducing oxidized Trx may facilitate Bcl-2 denitrosylation. (**III**) Under basal condition, Bcl-2 S-nitrosylation stabilizes protein structure and resists to the ubiquitin-proteasome degradation. Formation of heterodimers with proapoptotic protein such as Bax, inhibition of cytochrome c release and caspase protease family activation, and regulation of mitochondrial transmembrane potential are some of mechanisms by which Bcl-2 exerts its anti-apoptotic effect. (**IV**) In response to IL-24, Bcl-2 S-denitrosylation via both iNOS decrease (**a**) and TrxR1 increase (**b**) facilitates Bcl-2 ubiquitination, which finally is degraded by the 26S proteasome. Bax triggers release of cytochrome c and activation of caspase protease family, which mediated the intracellular proteolysis that is characteristic of cell apoptosis.

Although the expression of Bcl-2 is regulated by several mechanisms, such as transcription, posttranslational modification, dimerization and degradation [16], [17], increasing evidence demonstrates that posttranslational modification plays a critical role in a potential Bcl-2 turnover under stress condition [18], [19], [20]. Some studies indicate protein S-nitrosylation is a regulatory process in signal transduction pathways that adjusts the function of Bcl-2 by the covalent attachment of a nitric oxide (NO) group to a cysteine thiol side chain. It has been shown that the two cysteine residues of Bcl-2, Cys^158^ and Cys^229^ are responsible for S-nitrosylation of Bcl-2, and mutation of these two residues completely inhibit Bcl-2 S-nitrosylation [16]. S-nitrosylation has been regulated by NO synthases (NOSs) including neuronal NOS(nNOS), endothelial NOS(eNOS) and inducible NOS(iNOS) [21], [22]. Among three NO synthases, iNOS, a Ca^2+^-independent enzyme, is defined as the ‘high-output’ NOS, generating major amounts of NO. Some previous papers also show iNOS was found to be increased in advanced stages of melanoma and expression of MDA-7/IL-24 negatively regulated iNOS expression in malignant melanoma cell lines [23], [24], [25], suggesting that iNOS might contribute to enhance tumor progression. Nevertheless, the exact role of iNOS in tumorigenesis is unclear. Whether ZD55-IL-24-induced iNOS decrease would further influence Bcl-2 S-nitrosylation level is the first aim of our present study. Given that protein S-nitrosylation level not only depends on NO-mediated S-nitrosylation via NOS but also denitrosylating enzyme such as thioredoxin (Trx/TrxR) systems [26], we also investigate whether Bcl-2 S-nitrosylation reduction in response to ZD55-IL-24 is determined by both iNOS and Trx/TrxR systems.

Some present reports show that cisplatin-induced generation of reactive oxygen species causes Bcl-2 S-nitrosylation which inhibits 26S proteasome degradation, thus indicating that S-nitrosylation may exert the biological function through changing the protein stability. Similarly, NO-mediated S-nitrosylation of Bcl-2 associated with its ubiquitin degradation could be important in apoptosis resistance and the development of lung cancer induced by Cr(VI) and other carcinogens [16], [27]. Consequently, there is growing interest towards understanding the cellular mechanism whether Bcl-2 S-nitrosylation alteration in addition of ZD55-IL-24 is implicated in its ubiquitination and proteasomal degradation.

Since the mechanisms by which MDA-7/IL-24 suppresses Bcl-2 expression and facilitates cancer cell apoptosis have not been clarified. Our present study determined the significant role of Bcl-2 denitrosylation in its ubiquitin proteasome degradation, which finally mediates the caspase signal pathway activation and cancer cell apoptosis in response to ZD55-IL-24. Moreover, Bcl-2 S-nitrosylation diminishment in response to ZD55-IL-24 includes both iNOS-mediated S-nitrosylation and Trx/TrxR1 involved with denitrosylation, which subsequently facilitates the ubiquitin-mediated proteasome degradation. Such a tight relationship between Bcl-2 denitrosylation and ubiquitination sheds new light on IL-24-induced Bcl-2 degradation and specific tumor apoptosis.

## Materials and Methods

### Cells and Reagents

The human Henrietta Lacks (Hela) cell line, the human malignant melanoma cancer cell line (A375) and the human renal carcinoma cell line (7860) were obtained from Shanghai Cell Collection (Shanghai, China). Hela and A375 cells were cultured in Dulbecco's Modified Eagle's Medium (DMEM) (GIBCO BRL, Grand Island, NY) containing 5% fetal bovine serum (FBS, GIBCO-BRL), 2 mM L-glutamine, and 100 units/ml penicillin/streptomycin and a 5% CO2 environment at 37°C. The renal cancer 7860 cells were cultured in RPMI1640 medium, supplemented with 5% FBS and antibiotics. Sodium Nitroprusside (SNP), 2-(4-carboxyphenyl)-4,4,5,5-tetramethylimidazoline-1-oxy-3-oxide (PTIO), dithiothreitol (DTT), Dimethyl sulfoxide (DMSO) and Z-Leu-Leu-Leu-al (MG132) were purchased from Sigma-Aldrich Co (St. Louis, MO), Antibodies for Bcl-2, phospho-Bcl-2(Ser87), NOS2(iNOS), protein A-agarose, and IL-24-siRNA, iNOS-siRNA and scrambled control-siRNA were purchased from Santa Cruz Biotechnology, Inc. (Santa Cruz, CA). Antibodies for ubiquitin and S-nitrosocysteine were from Sigma-Aldrich Co (St. Louis, MO). Antibodies for β-actin, procaspase-3, cleaved caspase-3, procaspase-9, cleaved caspase-9 antibodies, anti-poly ADP-ribose polymerase (PARP) and cleaved PARP were purchased from Cell Signaling Technology, Inc. (Danvers, MA).

### Virus construction and production

pZD55, the E1B 55-kDa-deleted oncolytic adenovirus construction plasmid; pCA13, the E1-deleted adenovirus shuttle plasmid; and ZD55-enhanced green fluorescent protein (EGFP) with reporter gene EGFP were kindly provided by Professor Liu (Xin-Yuan Liu, Institute of Biochemistry and Cell Biology, Shanghai Institutes for Biological Sciences, Chinese Academy of Sciences, Shanghai, China). IL-24 was first cloned into pCA13 to form pCA13-IL-24. Then IL-24 excised from pCA13 was subcloned into pZD55 to construct pZD55-IL-24. Oncolytic adenovirus, ZD55-IL-24 was generated in HEK293 cells (Shanghai Cell Collection, Shanghai, China) by homologous recombination between pZD55-IL-24 and the adenovirus packaging plasmid pBHGE3 (Microbix Biosystems), respectively. Large scale purification of all adenovirus particles was performed by ultracentrifugation with cesium chloride according to standard techniques. The titers were determined using a plaque assay on HEK293 cells.

### Small interfering RNA assay

For experiments involving IL-24 specific siRNAs, cells (3×10^5^ per well) were plated in 6-well plates. At 24 h after incubation, Hela cells were washed, replenished with fresh medium and treated with ZD55-IL-24(20 MOI) for 12 h, cells were subsequently washed and placed in fresh culture medium and added with IL-24 specific siRNA (100 nM) by using a siRNA transfection reagent. After 24 h of siRNA transfection, cell lysates were prepared and subjected to the western blot analysis as described below.

For experiments involving iNOS-siRNAs, cells were transfected with 100 nM iNOS-specific siRNA for 12 h, cells were subsequently washed and placed in fresh culture medium and then treated with ZD-55-IL-24(20 MOI) for 12 h. After 24 h of siRNA transfection, cell lysates were prepared and subjected to the western blot analysis as described below.

### Western Blotting

After specific treatments, cells were incubated in lysis buffer containing 20 mmol/L Tris-HCl (pH 7.5), 1% Triton X-100, 150 mmol/L NaCl, 10% glycerol, 1 mmol/L Na_3_VO_4_, 50 mmol/L NaF, 100 mmol/L phenylmethylsulfonyl fluoride, and a commercial protease inhibitor mixture (Roche Molecular Biochemicals) for 20 minutes on ice. After insoluble debris was pelleted by centrifugation at 14,000 g for 15 minutes at 4°C, the supernatants were collected and determined for protein content using the Bradford method (Bio-Rad Laboratories, Hercules, CA). Proteins (80 µg) were resolved under denaturing conditions by SDS-PAGE (10%) and transferred onto nitrocellulose membranes. After blocking for 2 h in phosphate-buffered saline with 0.1% Tween20 (PBST) and 3% bovine serum albumin (BSA), membranes were incubated overnight at 4°C with the appropriate primary antibody in PBST containing 3% BSA. Membranes were then washed and incubated with alkaline phosphatase conjugated goat anti-rabbit IgG or anti-mouse IgG (Sigma, 1∶10 000) in PBST for 2 h and developed using NBT/BCIP color substrate (Promega, Madison, USA).

### Immunoprecipitation

For immunoprecipitation, cytosolic fractions (each containing 400 µg of proteins) were diluted four-fold with HEPES buffer containing 50 mM HEPES (pH 7.4), 150 mM NaCl, 10% glycerol, 1% Triton X-100, and 1 mM each of EGTA, EDTA, PMSF and Na_3_VO_4_. The samples were then pre-incubated for 1 h with 20 µl protein A agarose and centrifuged to remove any non-specifically adhered proteins from the protein A agarose. The supernatant was then incubated with 2 µg specific antibodies overnight at 4°C. After the addition of Protein A agarose, the mixture was incubated at 4°C for an additional 2 h. Samples were triple washed with HEPES buffer and eluted by sodium dodecyl sulfate-polyacrylamide gel electrophoresis (SDS–PAGE) loading buffer then boiled at 100°C for 5 minutes. Immune complexes were separated by 10% SDS-PAGE and analyzed by Western blotting as described above.

### Measurement of apoptosis by Annexin V analysis

An Annexin V-binding assay was used according to the manufacturer's instructions. Briefly, cells (3×10^5^ per well) in 6-well plates were treated with ZD55-IL24 (20 MOI) for the different time 12 h, 24 h, 36 h, 48 h, and 72 h. Cells were collected and the Annexin V-FITC and Propidium iodide (PI) dual-staining assay was performed according to the manufacturer's instructions ((Nanjing Keygen Biotech, China). Collected cells were briefly washed with ice-cold phosphate-buffered saline (PBS) twice and resuspended in 200 µl 1×binding buffer containing 5 µl Annexin V-FITC for 15 minutes and then in 300 µl 1×binding buffer containing 5 µl Propidium iodide (PI) for 5 minutes at room temperature in the dark. After incubation, the cells were analyzed using a FACStar flow cytometer.

### Cell viability assay

The carcinoma cells (1.0×10^4^ per well) were incubated in triplicate in a 96-well plate and treated with ZD55-IL-24 and NO modulators. Cell survival rate was evaluated by a standard 3-(4, 5-dimethylthazol-2-yl)-2,5-diphenyltetrazolium bromide (MTT) assay (Sigma, St. Louis, MO) in the presence or absence of the indicated test samples in a final volume of 0.2 ml for various lengths of time at 37°C. Thereafter, 20 µl MTT solution (5 mg/ml in PBS) was then added to each well. After 4 h incubation at 37°C, 150 µl DMSO was added. Finally the plates were shaken and the optical density at 570 nm was measured on ELX-800 spectrometer reader (Bio-Tek Instruments Inc., USA). Four replicate wells were tested per assay and each experiment was repeated three times. Percent cell viability was calculated as the ratio of the experimental samples to the control samples×100.

### Statistical analysis

Values are expressed as mean ±SD. Statistical analysis of the results was carried out using the Student's t-test or one-way analysis of the variance (ANOVA) followed by the Duncan's new multiple range method or Newman-Keuls test. *P*-values<0.05 were considered significant.

## Results

### Effect of ZD55-IL-24 on Bcl-2 expression and cancer cell viability

Protein expressions of IL-24 and E1A accompanied with adenovirus ZD55-IL-24 replication and translation in Hela, A375 and 7860 cells were detected by Western blotting at the different time points. Our data showed a distinct increase of IL-24 from 24 h to 72 h compared to controls, as shown in Fig. 1A. At the same time, E1A protein standing for replication ability of ZD55-IL-24 showed the obvious enhancement from 12 h to 72 h in Fig. 1B, which is similar to the time course of enhanced green fluorescence protein (EGFP) expression treated with ZD55-EGFP in Fig. 1D. In addition, Bcl-2 expression has the inverse decrease from 24 h to 72 h (Fig. 1C). Moreover, the Bcl-2 decline in response to ZD55-IL-24 was shown in a dose-dependent manner. The efficient titers of ZD55-IL-24 to inhibit the Bcl-2 expression are 10 and 20 MOI, as shown in Fig. 2A. To further investigate whether ZD55-IL24 affects three carcinoma cells survival, cell viability was determined by MTT assay (Fig. 1E). Our results showed that ZD55-IL-24 effectively decreased cell survival and this inhibition was also shown in a dose-dependent manner (Fig. 1E and 2C). Taken together, these results indicate the ZD55-IL24 could mediate a high-level and stable IL-24 expression from 24 h to 72 h and reduce Bcl-2 protein level in time- and dose-dependent manner.

### Effect of ZD55-IL-24 on Bcl-2 S-nitrosylation and ubiquitination

To investigate whether ZD55-IL-24 would contribute to alteration of Bcl-2 S-nitrosylation and ubiquitination, we detected Bcl-2 S-nitrosylation and ubiquitination level in Hela, A375 and 7860 cells. The results showed that ZD55-IL-24 in Hela cells diminished the Bcl-2 S-nitrosylation from 79% at 24 h to 38% at 48 h compared with the control group (Fig. 3A). In contrast, Bcl-2 ubiquitination increased from 1.4 fold at 24 h to 2.3 fold at 48 h, as shown in Fig. 3B. The results from A375 and 7860 cells were consistent with the above trend. To further confirm the potential role of IL-24 in the regulation of Bcl-2 S-nitrosylation and ubiquitination, specific IL-24-siRNA was used to knockdown IL-24 expression. Our data indicated IL-24-siRNA obviously restored Bcl-2 S-nitrosylation and thus suppressed Bcl-2 ubiquitination compared with scrambled control siRNA (Fig. 3C). Consequently, ZD55-IL-24 induced decreased Bcl-2 S-nitrosylation and increased Bcl-2 ubiquitination.

### Effect of ZD55-IL-24 on caspase activation and cancer cell apoptosis

To further determine whether the Bcl-2 aberrant diminishment in response to ZD55-IL-24 would result in the activation of caspase signal pathway in Hela cells, caspase-9, caspase-3 and PARP were detected at the different time points 12 h, 24 h, 36 h and 48 h respectively, as shown in Fig. 4A. The results demonstrated that procaspase-9 gradually decreased from 76% at 24 h to 36% at 48 h. In contrast, the cleaved caspase-9 correspondingly increased from 1.5- at 24 h to 2.5-fold at 48 h compared with the control group. Caspase-3 and PARP performed the similar alteration like caspase-9. Our results show that ZD55-IL-24 induced the cleavage of caspase-9, caspase-3 and PARP to activate the caspase signal pathway. In addition, Hela cells apoptosis was detected by Annexin V/PI and then analyzed using flow cytometry (Fig. 4B and C). The results indicated that ZD55-IL-24 dramatically enhanced apoptosis in Hela cells from 2.3- at 24 h to 7.4-fold at 72 h compared with the control group. Taken together, ZD55-IL-24 initiated the caspase signal pathway activation and cancer cell apoptosis.

### Effect of Bcl-2 S-nitrosylation alteration on the regulation of its ubiquitination in response to ZD55-IL-24

To investigate whether ZD55-IL-24 could regulate Bcl-2 S-nitrosylation via NO, which subsequently affects its ubiquitination, we treated Hela, A375 and 7860 cells with NO donor SNP, NO inhibitor PTIO and SNP co-administration of the reducing agent DTT after administration of ZD55-IL24, respectively. As shown in Fig. 5A, exogenous NO donor SNP effectively increased the Bcl-2 S-nitrosylation whereas this rescue effect was negated by co-administration with DTT. At the same time, NO inhibitor PTIO significantly reduced Bcl-2 S-nitrosylation. Conversely, NO donor SNP inhibited the ubiquitination of Bcl-2, but DTT co-treatment counteracted the effects of SNP. Moreover, NO inhibitor PTIO facilitated the ubiquitination of Bcl-2 in Fig. 5B. Fig. 5C shows ZD55-IL-24 treatment for 24 h caused a significant decrease of Bcl-2 expression, which was further decreased upon the addition of NO inhibitor PTIO. In contrast, treatment with NO donor SNP significantly resisted ZD55-IL-24-induced Bcl-2 down-regulation. Similarly, co-administration with DTT offset the Bcl-2 enhancement induced by SNP.

To further detect whether ZD55-IL-24-induced Bcl-2 degradation is ubiquitin-proteasome dependent, the proteasome inhibitor MG132 was use to block the proteasomal degradation in Hela, A375 and 7860 cells. Our results showed that MG132 significantly improved Bcl-2 expression (Fig. 5D) compared with vehicle control DMSO group, implicating that the ubiquitin-proteasome system was involved with Bcl-2 degradation induced by ZD55-IL-24. In summary, these results suggested that ZD55-IL-24 through its ability to regulate NO, may interfere with the Bcl-2 S-nitrosylation, promote its ubiquitination process and finally induce Bcl-2 proteasome dependent degradation. Previous evidence indicates that Bcl-2 phosphorylation induces a conformational change in Bcl-2, which controls its stability and apoptotic function. Dephosphorylation at Ser^87^ is the critical step of Bcl-2 degradation. To test whether NO-mediated S-nitrosylation would take part in the regulation of phosphorylation of Bcl-2 and therefore influence its ubiquitination and stability, we detected the effect of these NO modulator on Bcl-2 phosphorylation through Western blotting using specific phosphor-Bcl-2 (Ser^87^) antibody. The results showed that NO donors and inhibitors had no significant effect on the Bcl-2 phosphorylation level (Fig. 5E), suggesting that ZD55-IL-24 regulates Bcl-2 degradation via Bcl-2 S-nitrosylation/denitrosylation and ubiquitination in a phosphorylation-independent manner.

### Effect of Bcl-2 S-nitrosylation alteration on activation of caspase signal pathway and cancer cell apoptosis in response to ZD55-IL-24

Bcl-2, which is located on the outer mitochondrial membrane, is important for the suppression of mitochondrial manifestations of apoptosis. We examined whether alteration of Bcl-2 S-nitrosylation and ubiquitination in response to ZD55-IL-24 would subsequently contribute to activation of caspase-dependent pathway. Hela cells were treated with ZD55-IL-24 for 24 h and then added with NO donor SNP, NO inhibitor PTIO and SNP co-administration of DTT, respectively. Our data demonstrated that NO donor SNP attenuated the cleavage of caspase-9 and caspase-3. In contrast, NO inhibitor PTIO had the opposite effect and aggravated the activation of caspase-9 and caspase-3, as shown in Fig. 6A. Additionally, SNP inhibitor DTT co-administration reversed SNP protection of caspase-9 and caspase-3 from activation. It was shown that activation of caspase-9, caspase-3 was regulated by Bcl-2 S-denitrosylation induced by ZD55-IL-24. Moreover, we further assessed whether these NO modulators would influence apoptosis in Hela cells treated with ZD55-IL-24. Flow cytometric analysis of Annexin V/PI double stained cells was used to quantitatively assess apoptosis. Our data showed that incubation of ZD55-IL-24 for 48 h dramatically led to apoptosis increase in Hela cells, which was attenuated by NO donor SNP but aggravated by NO inhibitor PTIO. At the same time, SNP inhibitor DTT co-administration reversed the apoptosis inhibition of SNP. Moreover, proteasome inhibitor MG132 significantly alleviated apoptosis in Hela cells treated with ZD55-IL-24, indicating that inhibition of Bcl-2 degradation plays an important role in protection from caspase proteins cleavage and apoptosis in Hela cells (Fig. 6B and C). In addition, our results showed that NO donor SNP enhanced Hela cell survival. However, NO inhibitor PTIO or SNP co-administration with DTT diminished Hela cell survival, as shown in Fig. 6D. Taken together, these results suggested that alteration of Bcl-2 S-nitrosylation via NO modulators regulated Hela cell apoptosis in response to ZD55-IL-24.

### Effect of iNOS on Bcl-2 S-denitrosylation and ubiquitination in response to ZD55-IL-24

To explore whether Bcl-2 S-denitrosylation in response to ZD55-IL-24 was associated with iNOS-mediated S-nitrosylation, we detected the iNOS expression in Hela, A375 and 7860 cells, as shown in Fig. 7A. Our data showed that ZD55-IL-24 palpably and gradually diminished iNOS expression from 62% at 24 h to 36% at 48 h (Fig. 7A) in Hela cells, which is in agreement with the results from A375 and 7860 cells. In addition, specific iNOS-siRNA knocked down iNOS protein level (Fig. 7B), attenuated Bcl-2 S-nitrosylation level (Fig. 7D), subsequently enhanced Bcl-2 ubiquitination (Fig. 7E) and ultimately decreased Bcl-2 protein level (Fig. 7C), compared with the control siRNA, suggesting that iNOS was involved with Bcl-2 S-denitrosylation in response to ZD55-IL-24. Moreover, iNOS-siRNA also promoted activation of caspase signal pathway via Bcl-2 denitrosylation, as shown in Fig. 7F.

### Effect of TrxR1 on Bcl-2 S-denitrosylation and ubiquitination in response to ZD55-IL-24

Like phosphorylation and dephosphorylation, S-nitrosylation is reversible biological process. The extent of protein S-nitrosylation depends on the rate of both S-nitrosylation and denitrosylation. To explore whether the denitrosylation enzyme TrxR1 was implicated in Bcl-2 S-denitrosylation in response to ZD55-IL-24, we detected the time course of TrxR1 expression in Hela, A375 and 7860 cells, as shown in Fig. 8A. Our data showed that ZD55-IL-24 improved TrxR1 expression from 1.8-fold at 24 h after infection of ZD55-IL-24 to 2.1-fold at 48 h compared with the control group in Hela cells, which is consistent with results from A375 and 7860 cells. In order to further detect the role of TrxR1 in IL-24-coupled Bcl-2 S-denitrosylation, we treated these three cancer cells with TrxR1 inhibitor auranofin to detect the alterations of TrxR1 expression, Bcl-2 expression, Bcl-2 S-nitrosylation, and Bcl-2 ubiquitination. Fig. 8B–E showed that auranofin obviously diminished TrxR1 expression, restored Bcl-2 S-nitrosylation, down-regulated Bcl-2 ubiquitination, and enhanced Bcl-2 expression compared with vehicle control DMSO group. Additionally, auranofin also protected the caspase-9, caspase-3 and PARP from cleavage to activate caspase signal pathway, as shown in Fig. 8F. Taken together, these results suggest that ZD55-IL-24 induces Bcl-2 S-denitrosylation via regulation of TrxR1 and iNOS. Moreover, Bcl-2 S-denitrosylation facilitates its ubiquitin-proteasome degradation, then initiates the activation of caspase signal pathway and finally results in cancer cell apoptosis.

## Discussion

MDA-7/IL-24 that selectively kills cancer cells and decreases survival in adjacent tumor cells as a profound ‘bystander effect’ represents an appealing molecule for cancer gene therapy. Multiple reports confirm that IL-24 is mediated by mitochondrial pathways involving bcl-2 family gene members. Bcl-2 may regulate caspase activation through sequestration of unidentified caspase adaptors/activators and directly interact as substrates with different subsets of caspase [28]. In addition, overexpression of Bcl-2 can protect prostate cancer cells from apoptosis induced by MDA-7/IL-24, indicating that the caspase machinery activated by MDA-7/IL-24 in LNCaP cells might be preferentially inhibited only by Bcl-2 [29]. Our data also show that ZD55-IL-24 downregulates Bcl-2 expression and initiates the caspase signal pathway activation. However, the precise mechanism by which MDA-7IL-24 efficiently induces Bcl-2 decrease and cancer cell apoptosis remains to be defined.

The anti-apoptotic function of Bcl-2 is bound by its expression level tightly associated with some post-translational modifications. Protein S-nitrosylation is a cGMP-independent, redox-dependent modification that attaches nitrosonium ion (NO^+^) to cysteine sulfhydryls. Nitric-oxide synthases (NOS) play a role in the production of NO and may thus influence NO-mediated functions in tumor tissues. MDA-7/IL-24 expression in melanoma negatively correlates with inducible nitric oxide synthase (iNOS) expression. The present findings revealed that MDA-7/IL-24 treatment of melanoma cells activates phosphorylation of STAT3 and down-regulates interferon regulatory factor (IRF-1) whereas up-regulating IRF-2 expression, which reduces iNOS expression level [25], [30]. Our data also demonstrated that ZD55-IL-24 induced iNOS decrease in Hela, A375 and 7860 cells. Moreover, the inverse expression of MDA-7/IL-24 and iNOS is related with Bcl-2 S-nitrosylation. iNOS-siRNA dramatically inhibited Bcl-2 S-nitrosylation level and facilitated its subsequent ubiquitination degradation, thus activated the caspase signal pathway, suggesting that IL-24-mediated iNOS reduction is involved with regulation of Bcl-2 protein level via S-nitrosylation modification. However, like phosphorylation and dephosphorylation, S-nitrosylation and denitrosylation are the reversible post-translational modifications that implicate in regulation of the signal transduction [31], [32]. In the present study, we also demonstrate that Bcl-2 S-nitrosylation decrease in response to ZD55-IL-24 was attributed to not only inhibition of iNOS, but also activation of denitrosylation enzymes such as thioredoxin system. The thioredoxin system includes Trx protein, Trx reductase (TrxR) protein and NADPH. Trx denitrosylates S-nitrosylated protein through its dithiol moiety, thereby forming a reduced protein thiol (-SH) and oxidized Trx which is reduced by the seleno-flavoprotein Trx reductase (TrxR) and NADPH [33], as shown in Fig. 9, II. Some studies revealed that Trx system can reverse the inhibitory effects of NO on protein function by catalyzing protein denitrosylation [34]. However, inhibition of Trx1 or TrxR1 increased the amount of S-nitrosylated caspase-3, suggesting the cellular Trx system regulates basal and stimulus-induced caspase-3 denitrosylation [35]. In agreement with the previous studies, our results also showed that ZD55-IL-24 tilted the balance from Bcl-2 S-nitrosylation to denitrosylation through TrxR1 expression increase and iNOS protein level decrease. Moreover, TrxR1 inhibitor auranofin dramatically reinstated Bcl-2 S-nitrosylation via suppression of denitrosylating Trx1/TrxR1 system. Given that Fas or TNF-α as a trigger of cell apoptosis was associated with activation denitrosylation of some caspase isoforms and NF-kB [36], [37], whether MDA-7/IL-24 via the other modulator like Fas or TNF-α would directly or indirectly affect Trx1/TrxR1 system and be involved with Bcl-2 denitrosylation remains an outstanding issue.

Although the importance of gene expression in controlling apoptotic signal pathway has been underlined, whether Bcl-2 turnover under MDA-7/IL-24 is mainly determined by the post-translational modification is unclear. Our present findings verified that the proteasome inhibitor MG132 blocks the decrease of Bcl-2 protein induced by ZD55-IL-24, suggesting Bcl-2 degradation in response to IL-24 via ubiquitin-proteasome pathway. Some studies have shown that S-nitrosylation of Bcl-2 prevented its ubiquitin-proteasomal degradation and the apoptotic cell death induced by chromium (VI) in lung cancers [16]. Additionally, NO inhibited Bcl-2 ubiquitin-dependent degradation to restore Bcl-2 protein level in human lung carcinoma H-460 cells [27]. These studies demonstrated that Bcl-2 degradation induced by ubiquitination was tightly associated with S-nitrosylation modification. Although the exact mechanism by which Bcl-2 S-nitrosylation blocked Bcl-2 ubiquitination is not manifest, it may take part in the conformational change of S-nitrosylated Bcl-2 protein, which may prevent its recognition and subsequent attachment of ubiquitin by the enzyme ubiquitin ligase. Moreover, ubiquitin ligases themselves were identified as targets for S-nitrosylation, which inhibited these ligases like parkin, an E3 ubiquitin ligase [38]. Some previous studies showed that S-nitrosylation of protein, such as Akt/PKB, PLIP and caspases, has been reported to modulate their apoptosis activities [35], [39]. Our results also indicated that Bcl-2 down-regulation induced by ZD55-IL-24 was linked to Bcl-2 S-denitrosylation and subsequent Bcl-2 ubiquitination. On one hand, the peaks of Bcl-2 denitrosylation and ubiquitination were concurrent with the bottom of Bcl-2 expression. It suggested that Bcl-2 S-denitrosylation and ubiquitination modification were probably correlated incident. Moreover, IL-24-siRNA blocked Bcl-2 S-denitrosylation and ubiquitination to reverse the decrease of Bcl-2 expression, suggesting that IL-24 mediates the Bcl-2 S-denitrosylation and ubiquitin degradation. On the other hand, the addition of NO donor SNP inhibited Bcl-2 S-denitrosylation, which subsequent attenuated ubiquitination, whereas NO inhibitor PTIO showed the opposite effects. Additionally, a known inhibitor of S-nitrosylation DTT was able to prevent Bcl-2 S-nitrosylation, thus facilitated its ubiquitin degradation and activated the caspase protease family, indicating that NO was involved with regulation of Bcl-2 denitrosylation, which further influenced Bcl-2 ubiquitination and proteasomal degradation. Taken together, our present study suggested that ZD55-IL-24 induced Bcl-2 denitrosylation, which facilitated its ubiquitination and protein down-regulation through proteasome-mediated degradation.

Some reports show that phosphorylation of Bcl-2 had also been reported to affect the conformational changes and degradation via ubiquitination. Dephosphorylation at Ser^87^ by ROS and TNF-α has been shown to be required for ubiquitination and degradation of Bcl-2 [18]. However, our study demonstrated that Bcl-2 S-nitrosylation alteration via NO modulators was not involved with the phosphorylation of Bcl-2. Since NO played a crucial role in Bcl-2 denitrosylation and ubiquitination in cancer cell apoptosis induced by ZD55-IL-24, it suggested that Bcl-2 denitrosylation was a different mechanism from dephosphorylation induced by ROS. Nevertheless, protein S-nitrosylation has emerged as a redox-dependent post-translational modification. Alternatively, ROS probably exhibited effects on regulation of Bcl-2 stability through either denitrosylation or dephosphorylation. The detailed mechanism needed to be further investigated.

To detect whether IL-24 mediated Bcl-2 denitrosylation is involved with activation of the caspase signal pathway, we used pharmacologic manipulation such as SNP and PTIO to adjust Bcl-2 S-nitrosylation level and then detected the caspase protein expression in response to ZD55-IL-24. Our data revealed that Bcl-2 denitrosylation induced by ZD55-IL-24 triggered the activation of caspase-9, caspase-3, PARP and final carcinoma cell apoptosis from the results of western blotting and flow cytometry. In addition, NO donor SNP enhanced Bcl-2 S-nitrosylation and thus resisted cleavage of caspases. NO inhibitor PTIO had the opposite effect. Consequently, we deduced that Bcl-2 denitrosylaiton coupled with ubiquitination play an important role in activation of the caspase signal pathway in response to ZD55-IL24.

In summary, our study suggests an important, regulatory role of Bcl-2 stability in IL-24 mediated carcinoma cell apoptosis, as shown in Fig. 9. Under basal condition, Bcl-2 S-nitrosylation prevents it from ubiquitin degradation, which forms heterodimers with the proapoptotic protein Bax to neutralize its death effector properties and switch cancer cell to survival. In contrast, under stress condition, MDA7/IL-24 reduces Bcl-2 S-nitrosylation via down-regulation of iNOS and up-regulation of TrxR1, which further results in Bcl-2 ubiquitination modification. Released cytochrome c followed by Bcl-2 degradation promotes the caspase protease family activation, which mediates the intracellular proteolysis and finally induces carcinoma cell apoptosis. Seeing that increased iNOS production and Bcl-2 expression have been associated with several human tumors, this finding on the novel function of MDA-7/IL-24 on regulation of Bcl-2 denitrosylation may provide a valuable mechanism for MDA-7/IL-24 induced cancer-specific apoptosis.

## References

[1] Jiang H, Lin JJ, Su ZZ, Goldstein NI, Fisher PB (1995). Subtraction hybridization identifies a novel melanoma differentiation associated gene, MDA-7, modulated during human melanoma differentiation, growth and progression.. Oncogene.

[2] Jiang H, Su ZZ, Lin JJ, Goldstein NI, Young CSH (1996). The melanoma differentiation associated gene MDA-7 suppresses cancer cell growth.. Proc Natl Acad Sci USA.

[3] Fickenscher H, Hor S, Kupers H, Knappe A, Wittmann S (2002). The interleukin-10 family of cytokines.. Trends Immunol.

[4] Gupta P, Su ZZ, Lebedeva IV, Sarkar D, Sauane M (2006). MDA-7/IL-24: multifunctional cancer-specific apoptosis-inducing cytokine.. Pharmacol Ther.

[5] Su ZZ, Lebedeva IV, Sarkar D, Gopalkrishnan RV, Sauane M (2003). Melanoma differentiation associated gene-7, MDA-7/IL-24, selectively induces growth suppression, apoptosis and radiosensitization in malignant gliomas in a p53-independent manner.. Oncogene.

[6] Yacoub A, Mitchell C, Lebedeva IV, Sarkar D, Su ZZ (2003). MDA-7 (IL-24) Inhibits growth and enhances radiosensitivity of glioma cells in vitro via JNK signaling.. Cancer Biol Ther.

[7] Lebedeva IV, Su ZZ, Sarkar D, Kitada S, Dent P (2003). Melanoma differentiation associated gene-7, MDA-7/interleukin-24, induces apoptosis in prostate cancer cells by promoting mitochondrial dysfunction and inducing reactive oxygen species. Cancer Res..

[8] Lebedeva IV, Sarkar D, Su ZZ, Kitada S, Dent P (2003). Bcl-2 and Bcl-xL differentially protect human prostate cancer cells from induction of apoptosis by melanoma differentiation associated gene-7, MDA-7/IL-24.. Oncogene.

[9] Fisher PB (2005). Is MDA-7/IL-24 a “magic bullet” for cancer?. Cancer Res.

[10] Lebedeva IV, Sauane M, Gopalkrishnan RV, Sarkar D, Su ZZ (2005). MDA-7/IL-24: exploiting cancer's Achilles' heel.. Mol Ther.

[11] Lebedeva IV, Sarkar D, Su ZZ, Gopalkrishnan RV, Athar M (2006). Molecular target-based therapy of pancreatic cancer.. Cancer Res.

[12] Su Z, Lebedeva IV, Gopalkrishnan RV, Goldstein NI, Stein CA (2001). A combinatorial approach for selectively inducing programmed cell death in human pancreatic cancer cells.. Proc Natl Acad Sci U S A.

[13] Kluck RM, Bossy-Wetzel E, Green DR, Newmeyer DD (1997). The release of cytochrome c from mitochondria: a primary site for Bcl-2 regulation of apoptosis.. Science.

[14] Osford SM, Dallman CL, Johnson PW, Ganesan A, Packham G (2004). Current strategies to target the anti-apoptotic Bcl-2 protein in cancer cells.. Curr Med Chem.

[15] Liu XY, Gu JF (2006). Targeting gene-virotherapy of cancer.. Cell Res.

[16] Azad N, Vallyathan V, Wang L, Tantishaiyakul V, Stehlik C (2006). S-nitrosylation of Bcl-2 inhibits its ubiquitin-proteasomal degradation. A novel antiapoptotic mechanism that suppresses apoptosis.. J Biol Chem.

[17] Cory S, Adams JM (2002). The Bcl2 family: regulators of the cellular life-or-death switch.. Nat Rev Cancer.

[18] Breitschopf K, Haendeler J, Malchow P, Zeiher AM, Dimmeler S (2000). Posttranslational modification of Bcl-2 facilitates its proteasome-dependent degradation: molecular characterization of the involved signaling pathway.. Mol Cell Biol.

[19] Schinzel A, Kaufmann T, Borner C (2004). Bcl-2 family members: integrators of survival and death signals in physiology and pathology.. Biochim Biophys Acta.

[20] Kutuk O, Letai A (2008). Regulation of Bcl-2 family proteins by posttranslational modifications.. Curr Mol Med.

[21] Stamler JS, Lamas S, Fang FC (2001). Nitrosylation. the prototypic redox-based signaling mechanism.. Cell.

[22] Knowles RG, Moncada S (1994). Nitric oxide synthases in mammals.. Biochem J.

[23] Azad N, Iyer AK, Wang L, Lu Y, Medan D (2010). Nitric oxide-mediated bcl-2 stabilization potentiates malignant transformation of human lung epithelial cells.. Am J Respir Cell Mol Biol.

[24] Ekmekcioglu S, Ellerhorst J, Smid CM, Prieto VG, Munsell M (2000). Inducible nitric oxide synthase and nitrotyrosine in human metastatic melanoma tumors correlate with poor survival.. Clin Cancer Res.

[25] Ekmekcioglu S, Ellerhorst JA, Mumm JB, Zheng M, Broemeling L (2003). Negative association of melanoma differentiation-associated gene (MDA-7) and inducible nitric oxide synthase (iNOS) in human melanoma: MDA-7 regulates iNOS expression in melanoma cells.. Mol Cancer Ther.

[26] Hess DT, Matsumoto A, Kim SO, Marshall HE, Stamler JS (2005). Protein S-nitrosylation: purview and parameters.. Nat Rev Mol Cell Biol.

[27] Chanvorachote P, Nimmannit U, Stehlik C, Wang L, Jiang BH (2006). Nitric oxide regulates cell sensitivity to cisplatin-induced apoptosis through S-nitrosylation and inhibition of Bcl-2 ubiquitination.. Cancer Res.

[28] Grandgirard D, Studer E, Monney L, Belser T, Fellay I (1998). Alphaviruses induce apoptosis in Bcl-2-overexpressing cells: evidence for a caspase-mediated, proteolytic inactivation of Bcl-2.. EMBO J.

[29] Lebedeva IV, Sarkar D, Su ZZ, Kitada S, Dent P (2003). Bcl-2 and Bcl-x(L) differentially protect human prostate cancer cells from induction of apoptosis by melanoma differentiation associated gene-7, MDA-7/IL-24.. Oncogene.

[30] Ekmekcioglu S, Mumm JB, Udtha M, Chada S, Grimm EA (2008). Killing of human melanoma cells induced by activation of class I interferon-regulated signaling pathways via MDA-7/IL-24.. Cytokine.

[31] Mannick JB, Schonhoff CM (2002). Nitrosylation: the next phosphorylation?. Arch Biochem Biophys.

[32] Benhar M, Forrester MT, Stamler JS (2009). Protein denitrosylation: enzymatic mechanisms and cellular functions.. Nat Rev Mol Cell Biol.

[33] Benhar M, Forrester MT, Hess DT, Stamler JS (2008). Regulated protein denitrosylation by cytosolic and mitochondrial thioredoxins.. Science.

[34] Forrester MT, Foster MW, Stamler JS (2007). Assessment and application of the biotin switch technique for examining protein S-nitrosylation under conditions of pharmacologically induced oxidative stress.. J Biol Chem.

[35] Mannick JB, Hausladen A, Liu L, Hess DT, Zeng M (1999). Fas-induced caspase denitrosylation.. Science.

[36] Marshall HE, Stamler JS (2001). Inhibition of NF-kappa B by S-nitrosylation.. Biochemistry.

[37] Hoffmann J, Haendeler J, Zeiher AM, Dimmeler S (2001). TNFalpha and oxLDL reduce protein S-nitrosylation in endothelial cells.. J Biol Chem.

[38] Chung KK, Thomas B, Li X, Pletnikova O, Troncoso JC (2004). S-nitrosylation of parkin regulates ubiquitination and compromises parkin's protective function.. Science.

[39] Yasukawa T, Tokunaga E, Ota H, Sugita H, Martyn JA (2005). S-nitrosylation-dependent inactivation of Akt/protein kinase B in insulin resistance.. J Biol Chem.

